# Positions and Types of Pterion in Adult Human Skulls: A Preliminary Study

**DOI:** 10.4314/ejhs.v31i4.23

**Published:** 2021-07

**Authors:** Abebe Muche

**Affiliations:** 1 Department of Human Anatomy, School of Medicine, College of Medicine and Health Sciences, University of Gondar, Gondar, Ethiopia

**Keywords:** Frontozygomatic suture, inion, Pterion, sphenoid bone, zygomatic arch

## Abstract

**Background:**

A trauma to the skull in the area of the pterion usually causes rupture of the middle meningeal artery leading to life- threatening epidural hematoma. The objective of the study is to assess the prevalence of different types of pterion and to determine its location using valuable bony landmarks.

**Methods:**

On 90 dry adult human skulls of unknown sex, age and nationality the distance of different landmarks from pterion was measured using stainless steel sliding Vernier caliper. The data were analyzed using SPSS version-20 and an independent t-test analysis was implemented. A value of P< 0.05 was considered as statistically significant.

**Results:**

A higher occurrence of sphenoparietal type of pterion with the absence of frontotemporal type was noted. About 23% and 77% of the suture types are found to be unilateral and bilateral, respectively. There was a statistically significant difference between right and left sides of the skull in distances from the center of pterion to frontozygomatic suture, root of zygomatic arch, inion and in central thickness pterion.

**Conclusion:**

This study showed that the most prevalent type of pterion is sphenoparietal, and revealed asymmetry in the distances from center of pterion to frontozygomatic suture, root of zygomatic arch and inion, and its central thickness. Such findings could offer worthy information about the type and location of pterion, which could be relevant to anatomists, neurosurgeons, forensic medicine specialist and anthropologists.

## Introduction

The pterion is an H-shaped bony neurological landmark found at the junction of the frontal, sphenoid, parietal and the squamous part of temporal bone (1). It is located approximately 4 cm superior to the zygomatic arch and 3.5 cm posterior to the frontozygomatic suture (2).

Internally, the pterion is related to various anatomical structures, namely, the anterior division of the middle meningeal vessels, middle cerebral vessels, Sylvain fissure, circle of Willis, insula and Broca's motor speech area (on the left) and optic nerve (3).

Any traumatic blow to the pterion presumably causes rupture of the anterior divisions of the middle meningeal vessels causing an epidural haematoma subsequently resulting in compression of cerebral cortex and death unless proper intervention is carried out (4,5). Surgical approach via the pterion has been quoted as the most widely implemented approach for the proper management of intracranial anterior circulation aneurysm. This approach has better advantages over the traditional surgical approach with minor tissue damage, lesser brain retraction, a superior cosmetic results and a shorter duration of surgery (6).

According to Murphy (1956), pterion can be categorized into four types, namely, sphenoparietal, frontotemporal, stellate and epipteric suture (7). The sphenoparietal type is the most common suture formed by the articulation of the greater wing of sphenoid bone with parietal bone. The frontotemporal type is a pterional sutural pattern between the frontal and temporal bone. The stellate variety of suture is the site of articulation formed by the fusion of four flat bones, sphenoid, frontal, parietal and temporal bones. The epipteric type of pterion is characterized by the presence of small sutural bones between the sphenoid and parietal bones. The presence of epipteric or wormian (sutural) bone in the area, can possibly lead to wrong radiological diagnosis and clinical management of fracture in the pterion. The presence of sutural bones could possibly complicate surgical interventions involving burr hole surgeries as their extension may lead to orbital penetration (4,8,9).

Various evidences demonstrated that the type and location of pterion exhibits significant ethnic, sex and age-related variations (3,4,10–14). Even though such evidences exist, there is no documented information in Ethiopia so far. The present study is aimed to determine the type and location of the pterion using dry adult human skulls obtained from Northwest Ethiopia. The findings of this study could provide baseline information about the type and location of the pterion in the studied population that can be useful for anatomists, neurosurgeons, forensic pathologists and anthropologists.

The objective of the present study is to assess the prevalence of different types of pterion and to determine its location using valuable bony landmarks.

## Materials and Methods

A cross sectional study was conducted on ninety dried and intact adult skulls of unknown sex, age and nationality. The study was conducted from 20th of March to 20th of April, 2020 on dry skulls obtained from the anatomical museum in the Department of Human Anatomy, School of Medicine, College of Medicine and Health Sciences, University of Gondar, Ethiopia. Sutural patterns of the pterion and its distance from selected structural landmarks were assessed macroscopically on both sides. The data collection was performed after the ethical clearance and approval obtained from the School of Medicine Ethical committee, University of Gondar (Reference SOM 876/12 dated December 24, 2019). Skulls with any pathological deformities and trauma affecting the measurements, for instance fracture of zygomatic arch were excluded.

The sutural patterns of the pterion (sphenoparietal, frontotemporal, stellate and epipteric types) were studied on both sides of each skull using the principles of Murphy classification (7).

**Figure 1:**
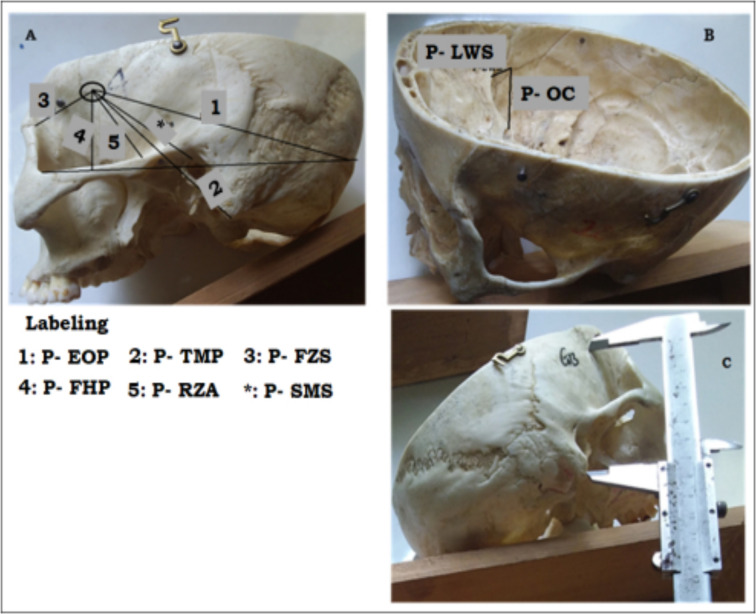
Representative picture presenting the distances from the pterion to some special external (A) and internal (B) structural landmarks and measurement from the central distance of pterion to various landmarks of skulls of unknown sex age and nationality obtained from the anatomical museum in the Department of Human Anatomy, School of Medicine, College of Medicine and Health Sciences, University of Gondar, Ethiopia using a Stainless steel Vernier caliper (C)

For the purpose of measurements of distances of various clinically important landmarks from the corresponding pterion, a circle of smallest radius was drawn just at the site of formation of the pterion. The center of the circle was considered as the center of pterion (Figure 1a and b). Distance measurements in centimeter were taken from the center of pterion with a stainless-steel sliding Vernier caliper (Figure 1c) twice and the average was taken as the actual measurement.

The following distance measurement parameters were taken on the lateral aspects of the skulls from the center of the pterion (P), 1) to the posterolateral aspect of the frontozygomatic suture (FZS), 2) to the root of zygomatic arch (RZA), 3) to the tip of the mastoid process (TMP), 4) to the suprameatal spine (SMS), 5) to the external occipital protuberance (EOP), 6) to the Frankfurt horizontal plane (FHP). Additionally, distance measurements were also taken from the internal aspect of the center of the pterion to the lateral end of the sphenoid ridge on the lesser wing of sphenoid bone (LWS) and to the lateral margin of the optic canal (OC). The thickness at the center of the pterion (TAAC) was also measured.

**Statistical analysis**: All the data were analyzed using SPSS version 20 statistical software. A comparison of the mean values between the sides was done using the independent t-test and a P-value less than 0.05 was considered as statistically significant. The data were presented as mean with the corresponding standard error of mean (SEM).

## Results

**Figure 2:**
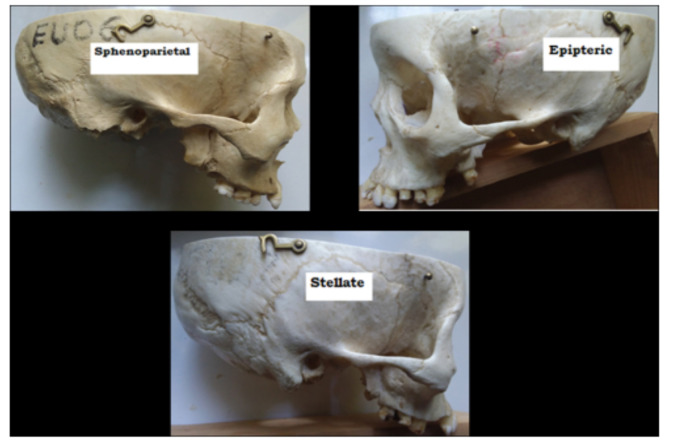
Identified types of the pteria on skulls of unknown sex age and nationality obtained from the anatomical museum in the Department of Human Anatomy, School of Medicine, College of Medicine and Health Sciences, University of Gondar, Ethiopia, 2020.

As it is presented in Table 1 and Figure 2, three types of pterion patterns (sphenoparietal, epipteric and stellate) were identified. Sphenoparietal was the most common type with frequency of 152 (84.4%), followed by epipteric 24 (13.3%). Frontotemporal type of pterion was not observed. In all the three identified types of pteria, there was asymmetric distribution.

**Table 1:** Distribution of types of pteria among skulls obtained in Northwest Ethiopia, 2020

Type of pterion	Right side	Left side	Both sides	Total
	Frequency	Frequency	Frequency	Frequency (%)
Sphenoparietal	14	6	132	152 (84.4%)
Frontotemporal	-	-	-	-
Epipteric	6	12	6	24 (13.3%)
Stellate	1	3	-	4 (2.2%)

In this study, the average distances between the center of pterion and several clinically and morphologically important structural landmarks were determined using the sliding stainless steel Vernier caliper. After checking for normality of distribution and homogeneity of variance, independent sample t-test was done to determine whether the central distance of the pterion from various bony landmarks differs with the sides of the skull as presented in Table 2. The mean distances from the center of pterion to FZS on the right side was 2.92 ± 0.05 cm and on the left side was 2.75 ± 0.05 cm. The distances were relatively shorter on the left side than on the right side and the difference was statistically significant (P= 0.021). However, the actual difference of the two sides was found to be small (Eta squared= 0.03). Similarly, the average distance from the RZA to the pterion on the right and left sides was 3.55 ± 0.04 cm and 3.30 ± 0.05 cm, respectively, and the difference was statistically significant (P= 0.000) with small actual difference between the two sides (Eta squared= 0.076).

**Table 2:** Comparison of the mean central distances from pterion (P) to various bony landmarks on the two sides

Bony Landmarks	Side	Mean	SEM	t	F	P-value	Eta squared
P-FZS	Right	2.919	.0534	2.33	1.92	.021*	.030
	Left	2.746	.0516				
P-RZA	Right	3.553	.0380	3.82	12.52	.000*	.076
	Left	3.302	.0537				
P-LWS	Right	1.693	.0233	-.98	8.60	.327	.005
	Left	1.733	.0334				
P-OC	Right	3.842	.0149	1.64	.39	.102	.015
	Left	3.801	.0201				
P-TMP	Right	7.690	.0459	.63	.12	.531	.002
	Left	7.649	.0466				
P-Inion	Right	12.518	.0669	-2.41	3.43	.017*	.031
	Left	12.726	.0546				
P-SMS	Right	4.968	.0380	-1.92	2.66	.057	.020
	Left	5.063	.0323				
P-EOP	Right	11.686	.0799	-1.60	5.90	.112	.014
	Left	11.847	.0614				
P-FHP	Right	3.491	.0439	1.76	1.63	.080	.017
	Left	3.387	.0399				
P-Lambda	Right	13.213	.1189	.28	.02	.784	.000
	Left	13.167	.1214				
P-TAAC	Right	.587	.0139	4.92	.24	.000*	.120
	Left	.491	.0136				

The distance between the center of pterion and inion was found to be 12.52 ± 0.07 cm on the right and 12.73 ± 0.05 cm on the left side being shorter on the right side as compared to the left side and the difference was statistically significant (P<0.05) but the effect of distance to the sides was small (Eta squared= 0.031) (Table 2). The central thickness of pterion was found to be 0.59 ± 0.01 cm and 0.41 ± 0.01 cm on the right and left sides, respectively. The left side of pterion was significantly thinner than the corresponding right sides (P<0.05, Eta squared=0.120) (Table 2).

## Discussion

Surgical approach via pterion has been cited as the most widely implemented approach to the proper management of intracranial anterior circulation aneurysmus. This approach has more advantages than the traditional surgical approach with minor tissue damage, less brain retraction, a superior cosmetic results and a shorter duration of surgery (6). Fundamentally, the knowledge of the pterion sutural patterns and its relationship to the bony landmarks is useful for specialist in various field of medical professions particularly for neurosurgeons (17, 18). The main findings of this study are notably the higher occurrence of the sphenoparietal type of pterion with the absence of frontotemporal type. About 23% and 77% of the suture types are found to be unilateral and bilateral, respectively. Furthermore, the central distances of the pterion to the FZS, RZA and inion, and the thickness at its center had statistically significant difference between the right and left sides of the skull.

The incidence of the different types of pteria among a diversified population of different countries were compared (1, 3, 4, 7, 8, 10–12, 14, 19–23) and illustrated in Table 3.

**Table 3:** Comparison of the percentage of pteria types with other population Type of pterion

Author	Population	Sample size	Sphenoparietal (%)	Frontotemporal (%)	Epipteric (%)	Stellate (%)
Murphy (1956)	Australian	368	73.23	7.75	18.34	0.68
Ersoy et al (2003)	Turkish	300	96	3.8	9	0.2
Oguz et al (2004)	Turkish	26	88	10	2	-
Mwachaka et al (2008)	Kenyans	50	66	15	12	7
Zalawadia et al (2010)	West India	42	91.7	2.4	4.8	1.2
Ilayperuma et al (2010)	Sri Lanka	52	74.04	4.81	21.15	-
Ma et al (2012)	New Zealandian	76	78.3	5.2	16.4	-
Ukoha et al (2013)	South East Nigerian	56	75.5	19.6	3.6	1.8
Sunday et al (2013)	Nigerian	62	86.1	8.3	-	5.6
Cimen et al (2019)	Middle and South	75	82	4.66	10.66	2.66
	Anatolian					
Kamath et al (2016)	India	72	79.25	10.25	6.3	4.2
Oguz et al (2004)	Turkish	26	88	0	2	10
Shenoy et al (2012)	India	75	77.33	0	21.33	1.34
Lee et al (2001)	Korean	149	76.5	0	40.3	0
Present Study	Unknown	90	70.6	0	15.6	2.8

Wang et al, (2006) explained the influence of environmental and/or genetic factors on the sutural patterns of pterion (13). A longitudinal study done using 368 Australian skulls reported four different stutural patterns of pterion (7). The incidence of the various types of pterion was 73.23%, 7.75%, 18.34% and 0.68% for sphenoparietal, frontotemporal, epipteric and stellate types, respectively. A study conducted in Turkey using 300 dried human skulls stated sphenoparietal type (96%), frontotemporal (3.7%), epipteric (9%) and stellate type (0.2%) (8). They revealed the existence of an epipteric or wormian bone at the pterion may complicate surgical orientation leading to complication during burr hole surgeries like orbital penetration. As it has been reported in another study on 26 dry adult skulls of Turks, 88% were sphenoparietal, 10% were frontotemporal and 2% were epipteric while the stellate type was absent (12). Different studies unanimously, reported that sphenoparietal type of pterion was found to be pre-dominant type of suture (3, 4, 10–12, 17, 20, 24). A study done on 52 dried adult Sri Lankan skulls reported the sphenoparietal type (74.04%) as the most common type followed by epipteric type (21.15%) and frontotemporal type (4.181%). Similarly, they did not find any stellate variety of pterion in their study (19). A Nigerian study also reported sphenoparietal type (77.33%), frontotemporal type (8.3%) and stellate type (5.6%) patterns of pterion without epipteric type (1). A study in India population showed sphenoparietal type (77.33%), epipteric type (21.33%), stellate type (1.34%) but a frontotemporal type of pterion was not seen (23). In another study done on 149 dried Korean skulls, the most frequent type of pterion found was sphenoparietal type (76.5%) followed by the epipteric type (40.3%) without the occurrence of stellate and frontotemporal types (20). In the present study, sphenoparietal (84.4%), epipteric (13.3%) and stellate (2.2%) types of pterion were identified. Of the observed patterns of pterion 23.3% and 76.7% were present unilaterally and bilaterally, respectively but no frontotemporal variety of pterion was observed. The presence or absence of frontotemporal pattern of pterion clearly indicates the contribution of genetic factor to the variation of occurrence ranged from zero in a British seventeenth century cementery to 9.8% in Nigerian crania (25).

The mean distance measurements between the center of pterion and different landmarks among different studies conducted elsewhere were compared (1, 3, 9–12, 14, 19, 21, 26, 27) and are depicted in Table 4. A study done on cone beam CR scans of 50 adult craniums and 76 adult dry skulls in New Zealand reported the significant clinical relationship of the anterior division of middle meningeal artery to the center of the pterion in the Frankfurt plane (21). As it was reported forty years ago the pterion is situated 3.0 – 3.5 cm posterior to the FZS (2). The mean distance of the center of the pterion from the posterolateral margin of the FZS in adult dry skulls was 3.1 ± 0.4 cm on the right side and 3.09 ± 0.4 cm on the left side (4). Studies conducted elsewhere reported a mean pterion to FZS distance ranging from 3.0 cm to 3.7 cm (10–12, 14, 27). However, Nigerian (3) and New Zealandian (21) studies stated shorter mean center of pterion to FZS distance on the right and left sides, respectively as (2.74 ± 0.17cm and 2.7 ± 0.06 cm; 2.6 ± 0.4 cm and 2.5 ± 0.4 cm). In the present study, the center of the pterion was found to be 2.92 ± 0.05 cm on the right side and 2.75 ± 0.05 cm on the left side above the posterolateral margin of the FZS (P<0.05, Eta squared= 0.03).

It has been reported that the center of the pterion is found to be 3 – 4 cm above the zygomatic arch (2). Similarly, studies conducted in a diversified population demonstrated the mean pterion - RZA distance in as presented in Table 4. In this study, the pterion was 3.55 ± 0.04 cm on the right side and 3.30 ± 0.05 cm on the left side superior to the RZA (P<0.05, Eta squared= 0.076).

The lesser wing of the sphenoid bone (LWS) is a common site for meningiomas and it can be approached through the pterional surgical technique. In this case, the distance between the internal portion of the pterion and the lateral margin of sphenoid bone is crucial. An Indian study done on 42 dry adult human skulls reported the mean distance between the pterion and LWS as 1.36 ± 0.35 cm on the right side and 1.33 ± 0.22 cm on the left side (14). Kenyans study reported that the distance of the pterion center from the lateral margin of LWS was 1.4 ± 0.33 cm on the right and 1.48 ± 0.32 cm on the left side far from the lateral margin of LWS (11). However, in the present study, the internal portion of pterion center was far from the lateral margin of LWS with a mean distance of 1.69 ± 0.02 cm on the right side and 1.73 ± 0.03 cm on the left side (P>0.05). The discrepancy of the mean distance is probably due to environmental and genetic variabilities.

Pterional approach is useful to reach to the optic canal containing optic nerve (CN II) and ophthalmic artery. In such a case, the distance measurements between the internal aspect of pterion and optic canal (OC) is decisive. Studies from India and Kenya reported that the internal surface of pterion center is far from the OC with a mean measurement of 4.52 ± 0.32 cm and 4.39 ± 0.4 cm on the right side and 4.37 ± 0.23 cm and 4.36 ± 0.4 cm on the left side, respectively (11, 14). In the present study, the mean measurement between the internal portion of pterion center and OC was 3.84 ± 0.01 cm on the right side and 3.80 ± 0.02 cm on the left side.

A very recent study done in Turkey using 75 dry adult skulls reported the mean measurement between the center of the pterion and the inion to be 13.55 ± 0.62 cm in male and 12.62 ± 0.63 cm in female (10). In another report based on skulls of male subjects of the Byzantine period, the distance between the pterion and the inion was found to be 13.80 ± 0.5 cm on the right side and 13.70 ± 0.40 cm on the left side (28). However, in this study, the mean measurement between pterion and inion was 12.52 ± 0.07 cm and 12.73 ± 0.05 cm on the right and left sides, respectively (P=0.017, Eta squared= 0.031).

A comparative study done on human skulls from 13^th^ to 20^th^ century using manual measurements revealed that the mean distance between pterion and the tip of mastoid process (TMP) in male subjects as 8.30 ± 0.34 cm on the right side and 8.50 ± 0.26 cm on the left side (28). In a study done in Turkey, the mean distance between pterion and TMP was found to be 8.02 ± 0.6 cm (10). On the other hand, in this study, the mean measurement between the center of the pterion and the TMP was 7.69 ± 0.05 cm on the right side and 7.65 ± 0.05 cm on the left side (P>0.05).

Cimen and collaborators (2019) measured the mean distance between pterion and external acoustic meatus as 5.71 ± 0.77 cm and 5.34 ± 0.36 cm in male and female skulls, respectively (10). In the present study, the measured mean distance between the center of pterion and the supra meatal spine was 4.97 ± 0.04 cm on the right side and 5.06 ± 0.03 cm on the left side (P>0.05). The difference may be due to geographical, genetic or methodological variations.

A study done in India on 100 dry skulls reported the mean thickness at the center of the pterion to be 0.352±0.145 cm (9). In addition, an Asian scholar reported mean different thicknesses at the center of the pterion as 0.513±0.167 cm in Thai skulls (26), 0.39 to 0.41 cm in Turks (12), and 0.319±0.085 cm in Korean skulls (29). In this study the central thickness of the pterion was 0.59 ± 0.01 cm on the right and 0.41 ± 0.01 cm on the left side (P<0.05, Eta squared= 0.12). Clinically, the knowledge of the thickness of pterion is very important for neurosurgeons which could be applied during internal and external neurosurgical fixation procedures.

In conclusion, according to the finding in this current study, sphenoparietal type of suture is the most frequent variety of pterion. The mean measurements between the center of pterion and FZS, RZA and inion and central thickness of the pterion had statistically significant difference between the right and left sides of the skulls. The findings of this study may, presumably, be useful for the anatomists, neurosurgeons, forensic pathologies and anthropologists in the area of the studied population. Further investigation on the skulls of identified sex, age and nationality, particularly Ethiopian skulls, using computed scan, X-ray and dry human skulls is strongly recommended.
